# Abstracts from the 13th WINFOCUS World Congress on Ultrasound in Emergency & Critical Care

**DOI:** 10.1186/s13089-017-0082-z

**Published:** 2017-12-21

**Authors:** Stephen Alerhand, Adam Nevel, Bret Nelson, Michael Halperin, Felipe Serrano, Gregor Prosen, Tjaša Banović, Stephanie J. Doniger, Mirjana Brvar, Barbara Furman, P. Gallego Rodríguez, Tomas Villén Villegas, A. Trueba Vicente, L. W. Alba Muñoz, C. Guillén Astete, N. Díaz García, N. García Montes, Jimena Areco, Daniel Terra, Fiorella Cavalleri, Siul Salisbury, Ana Rodríguez, Mohd Hashairi Fauzi, Zulaili Asri, Norainal Atiqah Mohamed, Mohmad Aswad Mohmad Amin, Adeline Marie Gnanasegaran Xavier, Mohd Anas Mohd Nor, Khairul Izwan Hashim, Shaik Farid Abdull Wahab, Mohd Boniami Yazid, Mohammad Zikri Ahmad, Ahmad Rasdan Ismail, Rohayu Othman, Mauro Constantini, Julio Pontet, Igor Sviridenko, Pablo Rodriguez, Christian Yic, Diego Méndez, Sylvia Noveri, Ana Soca, Mario Cancela, Pablo Rodriguez Luna, Rodrigo Martella, Silvina Fabretto, Erich Lidstone, Jacob Shapiro, Kristine Robinson, Cecilia Gómez Ravetti, Thiago Bragança Lana Silveira Ataide, Lidia Miranda Barreto Mourão, Nathália Costa Almeida Pinho, Lucas Vieira Chagas, Renan Detoffol Bragança, Vandack Nobre, Maria Thereza Meira Araujo, Luiz Ernani Meira Junior, Luciana Mendes, Jackson Andrade, Nayara Nobre Basso, Anna Cecília Castro e Abreu, José Muniz Pazeli Junior, Ana Luisa Silveira Vieira, Bernardo Costa Lemos, Marinna Marques Rodrigues Saliba, Maurício Dutra Costa, Pedro Andrade Mello, Rosimary Souza Vicentino, Juan Pablo Fernandez, Nicolas Ahualli, Humberto Insfran, Ivana Fatica, Jonatan Bornia, Paula Denardi, Ruben Daniel Algieri, Cristian Flores, Maria Soledad Ferrante, Gustavo Vassia, Carolina Brofman, Victor Ortiz, Elizabeth Krebs, Frances Shofer, Cameron Baston, Christy Moore, Wilma Chan, Anthony J. Dean, Nova Panebianco, Stefano Geniere Nigra, Carmela Graci, Vito Sgromo, Alberto Casazza, Giacomo Veronese, Miguel Montorfano, Giovanni Ricevuti, Marina Marazzi, María Fernanda Barbui, Gabriela Da Campo, Cecilia Ciarlo, Leonardo Vera, Matías Brizuela, Mariana Lía Brizuela, Marcos Aqcuavita, Javier Buchanan, José Alejandro Bujedo, Pablo Bravo Figueroa, V. Ricardo Carvajal, P. Oscar Bravo, N. Monserrat Navarro, J. Rodrigo Adasme, Carolina Méndez, Adi Osman, Azma Haryaty Ahmad, Seri Rohayu Neow Hanzah, Emilia Mohtar Razali

**Affiliations:** 1 Department of Emergency Medicine, Icahn School of Medicine at Mount Sinai, New York, NY 10029 USA; 2 Department of Emergency Medicine, Mount Sinai Hospital, 1 Gustave L. Levy Place, New York, NY 10029 USA; 3 Department of Emergency Medicine, Jacobi & Montefiore Medical Centers, Bronx, NY USA; 4 Center for Emergency Medicine Maribor, Slovenia, University of Maribor Medical Faculty, Maribor, Slovenia; 5 University of Maribor Medical Faculty, Maribor, Slovenia; 6 Department of Emergency Medicine, New York-Presbyterian, Brooklyn Methodist Hospital, Brooklyn, NY USA; 7 Dept. of Radiology, University Clinical Centre Maribor, Maribor, Slovenia; 8 Emergency Department, University Clinical Centre Maribor, Maribor, Slovenia; 9 Emergency Department, Hospital Universitario Ramon y Cajal, Madrid, Spain; 10 Surgery Division, Department of Anesthesiology, Thorax Institute, Montevideo, Uruguay; 11 Hospital de Clínicas, Universidad de la República, Asociación Española, Montevideo, Uruguay; 12 Emergency Ultrasound Unit, Department of Emergency Medicine, School of Medical Sciences, Universiti Sains Malaysia Health Campus, 16150 Kubang Kerian, Kelantan, Malaysia; 13 Department of Emergency Medicine, School of Medical Sciences, Universiti Sains Malaysia Health Campus, 16150 Kubang Kerian, Kelantan, Malaysia; 14 Department of Technology Creative and Heritage, Universiti Malaysia Kelantan, 16300 Bachok, Kelantan Malaysia; 15 Kolej Kemahiran Tinggi MARA, 17000 Pasir Mas, Kelantan Malaysia; 16 Hospital Privado de Comunidad, Mar del Plata, Argentina; 17 Intensive Care Unit Hospital Pasteur, Montevideo, Uruguay; 18 Intensive Care Unit Asociación Española Primera en Salud, Montevideo, Uruguay; 19 Department of Emergency Medicine, West Virginia University School of Medicine, Morgantown, WV USA; 20 West Virginia University School of Medicine, Morgantown, WV USA; 21 NIIMI (Núcleo Interdisciplinar de Investigação em Medicina Intensiva), Belo Horizonte, Brazil; 22 Hospital das Clinicas da UFMG, Belo Horizonte, Brazil; 23 Depto Clínica Médica da Faculdade de Medicina da, Universidade Federal de Minas Gerais, Belo Horizonte, Brazil; 24 Programa de Pós-graduação em Infectologia e Medicina Tropical, Universidade Federal de Minas Gerais, Belo Horizonte, Brazil; 25 Undergraduate Medical Student at Faculdades Integradas Pitágoras, Montes Claros, Brazil; 26 Departament of Emergency Medicine at Faculdades Integradas Pitágoras, Montes Claros, Brazil; 27 Departament of Radiology at Faculdades Integradas Pitágoras, Montes Claros, Brazil; 28 Barbacena’s School of Medicine, Minas Gerais, Brazil; 29 Hospital Aeronáutico Central-Fuerza Aérea Argentina, Buenos Aires, Argentina; 30 Division of Ultrasound, Department of Emergency Medicine, University of Pennsylvania, Philadelphia, PA USA; 31 University of Pavia, Pavia, Italy; 32 AREU-Azienda Regionale Emergenza Urgenza, Niguarda Hospital, Milan, Italy; 33 AREU-Azienda Regionale Emergenza Urgenza, Policlinico San Matteo Hospital, Pavia, Italy; 34 ASST-Azienda Socio-Sanitaria Terrritoriale Pavia-Ospedale Civile, Vigevano, Italy; 35 Emergency Medicine Resident, University of Bicocca, Milan, Italy; 36 Department of Ultrasound, Hospital de Emergencias “Dr. Clemente Álvarez” (HECA), Rosario, Argentina; 37 Department of Ultrasound and Doppler, Hospital de Emergencias “Dr. Clemente Álvarez” (HECA), Avenida Pellegrini 3205, Rosario, Argentina; 38 Unidad de Terapia Intensiva, Sanatorio Privado del Interior S.R.L, Rio Ceballos, Córdoba Argentina; 39 Unidad Paciente Crítico Pediátrico, Hospital San Juan de Dios, Santiago, Chile; 40 Respiratory Therapy Unit UC, Magister in Epidemiology, University of Los Andes, Las Condes, Chile; 41 Pediatric Intensivist, Pediatric Critical Patient Unit, San Juan de Dios Hospital, Santiago, Chile; 42 Neonatology Service, San Juan de Dios Hospital, Santiago, Chile; 43 Department of Trauma and Emergency Medicine, Raja Permaisuri Bainun Hospital, 30450 Ipoh, Perak Malaysia; 44 Department of Trauma and Emergency Medicine, National University Hospital, Cheras, 56000 Kuala Lumpur, Malaysia

## A1 Radial artery pseudoaneurysm diagnosed by point-of-care ultrasound five days after transradial catheterization: a case report

### Stephen Alerhand^1,2^, Adam Nevel^1,2^, Bret Nelson^1,2^

#### ^1^Department of Emergency Medicine, Icahn School of Medicine at Mount Sinai, New York, NY, 10029, USA; ^2^Department of Emergency Medicine, Mount Sinai Hospital, 1 Gustave L. Levy Place, New York, NY, 10029, USA

*Critical Ultrasound Journal 2017,* **9(Supp 1):**A1

**Background:** Though an extremely rare complication of arterial cannulation, the incidence of radial pseudoaneurysm may increase with the growing use of extended radial artery access for coronary angiography.

**Case report:** A 57 year-old female presented to the emergency department with painful swelling to the volar radial surface of her right wrist 5 days after a non-emergent transradial coronary angiography. An emergency physician used point-of-care ultrasound to diagnose a radial artery pseudoaneurysm. The high-frequency linear transducer allowed visualization of the arterial wall defect and connection between artery and hematoma on B-mode, turbulent pulsatile flow into the adjacent hematoma using color flow Doppler, and a to-and-fro waveform at the wall defect using spectral Doppler. Due to the size and characteristics of the pseudoaneurysm, as well as her pain and mild distal sensory deficits, it was determined that the patient required prompt operative repair.

**Discussion:** Bedside ultrasound is the most rapid and dynamic imaging modality for making diagnosing a radial artery pseudoaneurysm. Different techniques for treating this condition include conservative care, extended compression, thrombin injection, and surgery. Management primarily depends on the size of the pseudoaneurysm and its associated symptoms.

**Conclusion:** In addition to understanding the pathophysiology and risk factors for this condition, the emergency physician must be adept at using point-of-care ultrasound to both make the diagnosis and characterize its findings to determine management.

**Consent for publication:** The authors confirm that written informed consent was obtained for publication.

## A2 Type A thoracic aortic dissection suspected on resident performed bedside transthoracic echocardiography (TTE) in a patient with initial electrocardiogram (ECG) demonstrating an ST elevation myocardial infarction (STEMI)

### Michael Halperin, Felipe Serrano

#### Department of Emergency Medicine, Jacobi & Montefiore Medical Centers, Bronx, NY, USA

*Critical Ultrasound Journal 2017,* **9(Supp 1):**A2

**Case report:** A middle-aged male with no past medical history presented to the emergency department with chest pain, shortness of breath, nausea, and vomiting for 3 h. Distressed and diaphoretic, his vital signs were: blood pressure 92/53 mmHg, pulse 112, respirations 26/min, Sp02 96% on room air. His ECG was concerning for STEMI in the left circumflex territory and the catheterization team was activated in the middle of the night. Point of care TTE showed left ventricular dysfunction and a dilated aortic root with concern for an intimal tear just superior to the aortic valve, suspicious for aortic dissection. Point-of-care-ultrasound (POCUS) findings prompted the mobilization of the cardiothoracic surgery team. An aortogram showed a devastating type A aortic dissection involving the coronary arteries including a likely occlusion of the left main.

**Keywords:** Type A thoracic aortic dissection, Transthoracic echocardiogram (TTE), Point of care ultrasound (POCUS), ST elevation myocardial infarction (STEMI)

**Consent for publication:** The authors confirm that written informed consent was obtained for publication.

## A3 Survey of attendees of WINFOCUS USLS-BL course in Slovenia

### Gregor Prosen^1^, Tjaša Banović^2^, Stephanie J. Doniger^3^

#### ^1^Center for Emergency Medicine Maribor, Slovenia, University of Maribor Medical Faculty, Maribor, Slovenia; ^2^University of Maribor Medical Faculty, Maribor, Slovenia; ^3^Department of Emergency Medicine, New York-Presbyterian, Brooklyn Methodist Hospital, Brooklyn, NY, USA

*Critical Ultrasound Journal 2017,* **9(Supp 1):**A3

**Objective:** To assess the degree of uptake, regular use, and obstacles in applying point-of-care ultrasound (POCUS), following standardized WINFOCUS Ultrasound Life Support (USLS-BL) courses in Slovenia from 2009 to 2016.

**Methods:** An online survey of all WINFOCUS USLS-BL courses in Slovenia.

**Results:** Of 660 attendees, we obtained 125 complete responses (18.9%). Majority (67%) attendees were resident or junior attending physicians. Amongst specialties represented, attendees were from family medicine (34%), emergency medicine (19%), anaesthesia (11%), and internal medicine (13%). Majority of attendees (87%) evaluated course content and depth as appropriate. Following completion of course, 73% reported having access to US machines always or most of the time; 7% of respondents use POCUS more than 5 times daily while 44% use it at least “few times par day to few times per week”, and 14% have never used POCUS after the course. Following completion of the course, attendees used POCUS for the following applications: lung (74%), DVT (73%), AAA (71%), FAST (67%), and focused cardiac (62%). 42% felt “confident” about their FAST examinations, 41% of their AAA examinations, 41% of their lung US examinations and 22% of their focused cardiac examinations. Overall, 36% did not feel confident about any of their POCUS applications; 70% responded they didn’t keep up regular practice, mostly due to the lack of mentoring and/or continuous supervision.

**Conclusion:** Lack of continuous mentoring program and regular practice appear to be the is main obstacles to the widespread use and implementation of POCUS following a 2 day WINFOCUS USLS-BL introductory course in Slovenia.

## A4 Survey of a novel introductory POCUS course

### Gregor Prosen^1^, Tjaša Banović^2^, Mirjana Brvar^3^, Barbara Furman^4^, Stephanie J. Doniger^5^

#### ^1^Center for Emergency Medicine Maribor, Slovenia, University of Maribor Medical Faculty, Maribor, Slovenia; ^2^University of Maribor Medical Faculty, Maribor, Slovenia; ^3^Dept. of Radiology, University Clinical Centre Maribor, Maribor, Slovenia; ^4^Emergency Department, University Clinical Centre Maribor, Maribor, Slovenia; ^5^Department of Emergency Medicine, New York-Presbyterian, Brooklyn Methodist Hospital, Brooklyn, NY, USA

*Critical Ultrasound Journal 2017,* **9(Supp 1):**A4

**Objective:** This study sought to show that the basic level courses (WINFOCUS USLS-BL) give too much information to complete novice point-of-care ultrasound (POCUS) users to process, with little chance to consolidate knowledge and skill.

**Methods:** We divided traditional 2 day USLS-BL course structure into two separate 1-day courses, conducted at 5 months apart. Prior to initiation of Day 1/introductory course, attendees were required to view 4+ hours of introductory videos on the basic POCUS applications. The course consisted of short review didactic lectures followed by hands-on practice of each application; measurement of abdominal aorta, the identification of a proximal DVT, the FAST exam including SC4C view of heart, the identification of lung sliding and haemothorax, and the assessment of IVC collapsibility. Following the course, all attendees (n = 22) were sent an online survey (10-point Likert-like scale) in order to assess their opinions and satisfaction regarding the course materials and format.

**Results:** Post-course survey response rate was 86%. Attendees were satisfied with the pre-course videos (8.8). Prior to viewing the videos and taking the course, attendees graded their level of knowledge and skill at 3.6. After the course and after acquiring their first independent POCUS image acquisitions, attendees’ average confidence level was 6.5. The attendees’ overall course assessment was 9.2.

**Conclusion:** Attendees of a 1-day introductory POCUS course were highly satisfied with pre-course materials (flipped classroom) and overall course structure. Attendees felt reasonably confident to start acquiring images independently, especially in conjunction with an online mentoring program.

## A5 Wide-QRS tachycardia + shock: ruptured AAA-importance of the RUSH protocol in the ER

### P. Gallego Rodríguez, Tomas Villén Villegas, A. Trueba Vicente, L. W. Alba Muñoz, C. Guillén Astete, N. Díaz García, N. García Montes

#### Emergency Department, Hospital Universitario Ramon y Cajal, Madrid, Spain

*Critical Ultrasound Journal 2017,* **9(Supp 1):**A5

**Case report:** 85-year-old male, with a history of chronic AF, who is admitted to the ER because of syncope while at his health center. A wide-QRS tachycardia at 250 bpm was detected, calling the Prehospital Emergency Unit (SUMMA) who administered Adenosine unsuccessfully, so a bolus of Amiodarone was administered. Upon arrival he presented AF with a controlled ventricular response, but hypotension around 70/40 so they began aggressive IV fluid therapy. Despite the good response in the BP with the administration of only 300 cc of normal saline, and given the unexplained initial hypotension, a RUSH protocol ultrasonography was performed, detecting an abdominal aorta aneurism with a diameter of 9 cm. An urgent CT scan was requested, confirming the existence of a 9 cm in diameter infrarenal aorta aneurism with an adjacent left perirenal complicated hematoma. The vascular surgeons were warned, deciding the performance of emergency surgery and inserting a right aortoiliac endoprosthesis. The patient had to undergo a second surgery because of an endoleak. Good progress, being discharged 6 days later.

**Conclusions:** This case demonstrates the importance of PoCUS when evaluating a patient in shock. It also comes to prove the need of providing all health resources with ultrasonography equipment, given the fact that despite all the published evidence, its use is not yet widespread.

**Consent for publication:** The authors confirm that written informed consent was obtained for publication.

## A6 Lung blockage assessment through ultrasound in thorax surgery: first experience in our medium

### Jimena Areco^1,2^, Daniel Terra^1,2^, Fiorella Cavalleri^1,2^, Siul Salisbury^1,2^, Ana Rodríguez^1,2^

#### ^1^Surgery Division, Department of Anesthesiology, Thorax Institute, Montevideo, Uruguay; ^2^Hospital de Clínicas, Universidad de la República, Asociación Española, Montevideo, Uruguay

*Critical Ultrasound Journal 2017,* **9(Supp 1):**A6

**Goal:** Assessing the validity and effectiveness of pulmonary ultrasound against clinical method to corroborate left selective intubation on thorax surgery.

**Materials and methods:** Transversal study, observational, prospective, double blind. 59 patients in 2 different stages where included: (1-n 15 technique development; 2-n 44). After intubation with left double-lumen tube, sequential clamping of both lights, both clinically assessment of position and through ultrasound with subsequent confirmation through fibrobronchoscopy (reference standards)

*Stage 2 results:* In 56.8% (n = 25) of cases the tube was placed properly.

Ultrasound validation (proper collocation): sensitivity of 84.0% (IC 95% 63.1–94.7), specificity of 94.7% (IC 95% 71.9–99.7), positive predictive values 95.4% (IC 95% 75.1–99.7), negative predictive value 81.8% (IC at 95% 59.0–94.0). Validity of pulmonary auscultation: sensitivity of 96.0% (IC at 95% 77.7–99.8), specificity of 100% (IC at 95% 79.1–100), positive predictive values of 100% (IC at 95% 82.8–100), negative predictive value of 95% (IC at 95% 73.1–99.7).

**Discussion:** The difference in results with other authors might respond to difference in expertise (first experience on our medium), wider inclusion criteria, and number of patients. We propose increasing the “n” and adding other ultrasonic signs of assessment.

**Conclusion:** Ultrasound is presented in a promising way as a complementary tool to clinic evaluation.

## A7 Sonographic abdominal A-lines could suggest pneumoperitoneum on bedside ultrasound: don’t miss it!

### Mohd Hashairi Fauzi, Zulaili Asri, Norainal Atiqah Mohamed, Mohmad Aswad Mohmad Amin

#### Emergency Ultrasound Unit, Department of Emergency Medicine, School of Medical Sciences, Universiti Sains Malaysia Health Campus, 16150 Kubang Kerian, Kelantan, Malaysia

*Critical Ultrasound Journal 2017,* **9(Supp 1):**A7

**Objective:** To demonstrate the use of bedside ultrasound in detecting intraperitoneal free gas in acute abdomen patient presented to Emergency Department (ED)

**Methods:** A 78-year-old lady presented to ED with history of progressively worsening abdominal pain and vomiting for a week. She had multiple medical problems including diabetes mellitus and hypertension. On arrival, she was lethargy but arousable to pain. Her vitals were normal except for low blood pressure (90/60 mmHg) and slight tachycardia (110 bpm). Her abdomen was distended and tender over the epigastric area. Initial erect chest radiograph was inconclusive. Bedside abdominal ultrasound then was performed using low frequency probe and revealed a free fluid in Morrison pouch and prehepatic area with presence of multiple, equally space, horizontal, hyper echoic lines repeating down the screen which resembles A-lines in thoracic ultrasound. Repeated erect chest radiograph later confirms pneumoperitoneum. Exploratory laparotomy was done and revealed perforated gastric ulcer 2.5 cm at anterior body of stomach.

**Results:** Perforated viscus is not common yet required urgent intervention. Although plain radiograph is always the first line imaging in patient suspected perforation, ultrasonography can usually show the signs of pneumoperitoneum if present. The main sonographic signs are increased echogenicity of peritoneal stripe and multiple reflection artifacts resemble A-lines.

**Conclusion:** Ultrasound users should aware of these signs when performing the point-of-care ultrasound (POCUS) in suspected perforation patient presented to ED

**Consent for publication:** The authors confirm that written informed consent was obtained for publication.

## A8 Case report mediastinal mass mimicking lung hepatization: the role of bedside ultrasound

### Mohd Hashairi Fauzi, Adeline Marie Gnanasegaran Xavier, Mohd Anas Mohd Nor, Khairul Izwan Hashim, Shaik Farid Abdull Wahab, Mohd Boniami Yazid, Mohammad Zikri Ahmad

#### Department of Emergency Medicine, School of Medical Sciences, Universiti Sains Malaysia Health Campus, 16150 Kubang Kerian, Kelantan, Malaysia

*Critical Ultrasound Journal 2017,* **9(Supp 1):**A8

**Objective:** To demonstrate the use of bedside ultrasound in detecting mediastinal mass in Emergency Department (ED).

**Methods:** We presented a case of 18-year-old male came to ED with complaints of fever, cough, shortness of breath and pleuritic chest pain. Examination revealed a febrile, tachypneic patient with chest examinations suggestive of left pleural effusion. Full blood count showed leukocytosis and thrombocytopenia and the chest radiograph showed massive left sided pleural effusion. However, bedside ultrasound showed presence of lung hepatization with hypoechoic lesion at anterior zone of both lungs which was atypical of lung collapse-consolidation. Urgent CT thorax was proceed and confirmed the lesion visualized on ultrasound as mediastinal lymphoma.

**Results:** A rapidly progressive symptoms should trigger the suspicion of malignancy even in the young healthy group of individuals. Between pulmonary infection and mediastinal mass, the clinical features may overlap, but the use of ultrasound helps to distinguish between these two pathologies. Sonographic signs that suggestive of mediastinal mass include lack of pleural lines and sliding sign, absence of A-profile and homogeneity difference of the lesion. This helps to decide the urgency of doing CT thorax and initiate the necessary treatment as the management greatly varies.

**Conclusion:** The use of lung ultrasound in ED is important in detecting undiagnosed mediastinal mass. It helps not only to rule out differential diagnosis, but it also helps to increase a suspicion based on initial clinical findings and laboratory parameters.

**Consent for publication:** The authors confirm that written informed consent was obtained for publication.

## A9 Transesophageal ECHO: an ergonomic point of view

### Shaik Farid Abdull Wahab^1^, Ahmad Rasdan Ismail^2^, Rohayu Othman^3^, Mohd Hashairi Fauzi^1^, Mohd Boniami Yazid^1^, Mohammad Zikri Ahmad^1^

#### ^1^Emergency Ultrasound Unit, Department of Emergency Medicine, School of Medical Sciences, Universiti Sains Malaysia Health Campus, 16150 Kubang Kerian, Kelantan, Malaysia; ^2^Department of Technology Creative and Heritage, Universiti Malaysia Kelantan, 16300, Bachok, Kelantan, Malaysia; ^3^Kolej Kemahiran Tinggi MARA, 17000, Pasir Mas, Kelantan, Malaysia

*Critical Ultrasound Journal 2017,* **9(Supp 1):**A9

**Objective:** To evaluate the risk of musculoskeletal disorder (MSD) among medical officers that perform transesophageal echocardiogram (TEE) at Accident and Emergency Department, Hospital Universiti Sains Malaysia.

**Methods:** Rapid entire body assessment (REBA) is use to determine the risk level of MSD among the medical officers. The voluntarily based participants had been assigned to perform the procedure. For each of the participants, their body postures were captured using high resolution camera. The participants body postures were evaluated and recorded throughout the procedure. The risk of MSD for each participant were calculated in order to determine whether the risk of MSD is, negligible, low, medium, high or very high.

**Results:** A total of 10 medical officers took part in this study. Finding shows the medical officers that conduct TEE face high risk of MSD due to the poor body posture during the procedure. REBA score starting from 9 shows the medical officers is categorized under high risk of MSD. Prolonged probe holding, twisted wrist and body of the medical officers contributed to the high REBA score. Frequent wrist movement in less than 1 min also contribute to the high score.

**Conclusion:** The use of TEE in accident and emergency department is becoming an important procedure to assist in diagnosis and saving patients’ life. However, the risk of MSD due to this procedure affect the medical officer’s health and should be given serious attention.

## A10 Is performing FAST causing musculoskeletal injury?

### Shaik Farid Abdull Wahab^1^, Ahmad Rasdan Ismail^2^, Rohayu Othman^3^, Mohd Hashairi Fauzi^1^, Mohd Boniami Yazid^1^, Mohammad Zikri Ahmad^1^

#### ^1^Emergency Ultrasound Unit, Department of Emergency Medicine, School of Medical Sciences, Universiti Sains Malaysia Health Campus, 16150 Kubang Kerian, Kelantan, Malaysia; ^2^Department of Technology Creative and Heritage, Universiti Malaysia Kelantan, 16300, Bachok, Kelantan, Malaysia; ^3^Kolej Kemahiran Tinggi MARA, 17000, Pasir Mas, Kelantan, Malaysia

*Critical Ultrasound Journal 2017,* **9(Supp 1):**A10

**Objective:** To determine the risk of musculoskeletal disorder (MSD) among medical officers that conduct focus assessment with sonography in trauma (FAST).

**Methods:** Rapid entire body assessment (REBA) is used to evaluate the level of risk of MSD among the medical officer. The score for REBA are range from 1, 2 to 3, 4 to 7, 8 to 10 and 11 and above. Each score represents different risk level of MSD. This study has been done at Accident and Emergency Department, Universiti Sains Malaysia, Kelantan, Malaysia. During this study, 30 medical officers, had performed FAST on patients. The medical officers involved in this study are based on voluntarily basis. The body postures of each of the medical officers were observed and recorded using camera. REBA score was calculated based on their postures.

**Results:** Finding shows the risk of musculoskeletal disorder among medical officers that conducted FAST did exist. REBA scores show the risk of musculoskeletal disorder, range from 4 to 9. According to REBA score, 4–7 is in medium risk, thus further investigation is needed to improvise the condition of the body posture. REBA score 9 falls under high risk to develop MSD, therefore indicates immediate further investigation and relevant changes need to be done.

**Conclusion:** FAST has been increasingly done in accident and emergency department. Since the finding revealed an alarming risk of MSD, it is important to address this matter appropriately. This is essential in developing a safe working approach for medical officers that conduct the procedure.

## A11 Accessibility to echocardiographic intraoperative transthoracic windows in abdominal surgery under general anesthesia

### Mauro Constantini

#### Hospital Privado de Comunidad, Mar del Plata, Argentina

*Critical Ultrasound Journal 2017,* **9(Supp 1):**A11

**Background and objective:** There is enough bibliography that supports the use of transthoracic echocardiography (TTE) and the impact it generates in the field of critical medicine. However, the main limitation in anesthesia is access to windows in patients with unchanged positions and usually in mechanical ventilation. Therefore we decided to conduct an observational study regarding the obtaining of basic echocardiographic windows in patients under general anesthesia.

**Methods:** 50 patients were enrolled. After ventilator setting, in dorsal decubitus and with the surgical fields placed, 4 windows were explored: subcostal, apical, parasternal and supraesternal. PEEP, tidal volume, BMI, age, sex were recorded. Each window was evaluated with a score: 2 points: optimal, 1 point: partial, 0 point: the window can not be obtained.

**Results:** The subcostal window could not be obtained in any patient (surgical fields). The remaining 3 windows on score of 6 possible points the average was 5.1. The parasternal window obtained a mean of 1.3 points. The most frequent cause of impossibility of access to the window was the presence of the lung. There was no difference between subgroups (PEEP > 10 and BMI > 30) and score obtained.

**Conclusion:** The accessibility to the apical and suprasternal windows was close to optimal. The parasternal window had smaller scores but had no relation to the level of PEEP nor the BMI. Limitations: small number of patients and very limited shelter to extrapolate findings.

## A12 Impact of systematic point-of-care ultrasound (POCUS) on admission to Intensive Care Unit (ICU)

### Julio Pontet^1,2^, Igor Sviridenko^1^, Pablo Rodriguez^1^, Christian Yic^2^, Diego Méndez^2^, Sylvia Noveri^1,2^, Ana Soca^1^, Mario Cancela^2^

#### ^1^Intensive Care Unit Hospital Pasteur, Montevideo, Uruguay; ^2^Intensive Care Unit Asociación Española Primera en Salud, Montevideo, Uruguay

*Critical Ultrasound Journal 2017,* **9(Supp 1):**A12

**Objective:** Measure the POCUS impact on use of resources, morbi-mortality, diagnoses and therapeutic decisions.

**Methods:** A prospective controlled-study, in two ICUs with assignment to two groups: “US-group” with systematic ultrasound examination of the optic nerve, thorax, heart, abdomen, venous system, performed at the bedside by trained Intensivist. Another “control group” was formed with patients attended by Intensivists who did not perform ultrasound. Approved by Ethics Committee. Informed consent was obtained.

**Results:** We included 72 patients, 36 in each group, without differences in age, sex, APACHE II score, or reason for admission. To 5 days of admission, there was less utilization of resources in the US group vs control per patient: chest radiology (2.6 ± 2.0 vs 4.1 ± 3.5, p = 0.01), ultrasound by specialist (0.6 ± 0.7 vs 1.1 ± 0.7, p = 0.003), computed tomography (0.5 ± 0.6 vs 0.9 ± 0.7, p = 0.01). The delay to perform ultrasound was 2.1 ± 1.6 h vs 7.7 ± 6.7, p = 0.0001. The water balance (WB) was more negative in the US-group at 48 and 96 h (p = 0.01). There was significant correlation between WB and LVEF (r = 0.6). The time of mechanical ventilation was lower in US-group (5.1 ± 5.7 days vs 8.8 ± 9.4) p = 0.04. Mortality was similar. In the US-group there was a change of diagnosis in 12 cases (33%), pharmacological changes in 15 (42%) and interventional maneuvers in 7 (19%).

**Conclusions:** Systematic POCUS determines the lesser use of other diagnostic resources and shorter time of mechanical ventilation, possibly due to greater accuracy in the treatment of blood volume.

## A13 Experience in ultrasonic-guided central line placement in Intensive Care Unit (ICU)

### Julio Pontet, Pablo Rodriguez Luna, Igor Sviridenko, Rodrigo Martella, Silvina Fabretto, Ana Soca

#### Intensive Care Unit, Hospital Pasteur, Montevideo, Uruguay

*Critical Ultrasound Journal 2017,* **9(Supp 1):**A13

**Background:** Even though advantages of ultrasound line placement seem obvious, in our environment recently has begun its use, and only part of the Intensivists perform it.

**Objective:** This study aims to compare the degree of difficulty and the incidence of complications in central venous line placement, with or without ultrasound.

**Methods:** Prospective controlled study comparing 125 line placement in 105 patients, 55 with ultrasound (US-group) and 70 with landmark guided techniques (blindly, control-group). 121 placement (97%) were performed by resident physicians.

**Results:** The majority of accesses were via jugular in both groups, but with 81% anterior jugular access in the US-group vs. 88% of posterior jugular in control-group (p = 0.0001).

Difficulties with 3 or more punctures were: 1 case in US-group (2%) vs 23 in the control (32%) p = 0.0001. There were no complications in the US-group, while in the control group there were 14 (20%): hematomas 6 (9%), arterial puncture 7 (10%), pneumothorax 1 (1.4%), all of which were by residents of the second or third year. There was also no relationship with the experience of the operator according to the group, since only a single eco-guided access was made by resident of the first year (non-expert). The frequency of obesity was similar for both groups. There were no cases of catheter-related bacteriemia.

**Conclusions:** Real-time ultrasonic-guided central line placement in ICU was associated with less difficulty and absence of complications that were presented with landmark guided techniques.

## A14 Early recognition of left ventricular aneurysm with point-of-care ultrasound

### Erich Lidstone^1^, Jacob Shapiro^2^, Kristine Robinson^1^

#### ^1^Department of Emergency Medicine, West Virginia University School of Medicine, Morgantown, WV, USA; ^2^West Virginia University School of Medicine, Morgantown, WV, USA

*Critical Ultrasound Journal 2017,* **9(Supp 1):**A14

**Background:** Left Ventricular aneurysm (LVA) is one of several diagnoses to consider when evaluating chest pain in the emergency department (ED), with electrocardiogram (ECG) findings that are potentially similar to acute ST-elevation myocardial infarction (aSTEMI). Point-of-care ultrasound (PoCUS) facilitates in establishing the diagnosis, preventing unnecessary activation of the catheterization laboratory or administration of thrombolytics.

**Objective:** We present an LVA case, illustrating classic ECG findings and sonographic changes.

**Case report:** A 79-year-old female with a remote history of myocardial infarction and percutaneous intervention presented to the ED with worsening, diffuse chest pain. Her ECG demonstrated an old infarct, but no acute ischemia. Classic ECG findings for LVA include STEMI in leads V1–V4 with well-established Q-waves and T-wave inversions in the lateral leads. Additionally, T-wave amplitude is typically lower than the hyperacute T-waves seen in aSTEMI, and the ratio of T-wave to QRS amplitude (TW/QRS) is less than 0.36 in V1–V4. The patient’s physical exam was unremarkable. PoCUS echocardiogram was performed using a SonoSite X-porte ultrasound machine with a phased array probe. An impressively large LVA impeding the right ventricular function was visualized. A computed tomography thorax with contrast re-demonstrated the 8.7 by 8.5 cm apical LVA. The patient was admitted for further intervention but subsequently refused surgery and was discharged in stable condition.

**Discussion:** POCUS is an effective tool in diagnosing LVA to expedite clinical decision making, especially in cases where the ECG findings mimic aSTEMI.

**Consent for publication:** The authors confirm that written informed consent was obtained for publication.

## A15 Use of lung ultrasound (LUS) to predict invasive mechanical ventilation requirement in patients with onco-hematology diseases: a pilot study

### Cecilia Gómez Ravetti^1,2,3^, Thiago Bragança Lana Silveira Ataide^1,2^, Lidia Miranda Barreto Mourão^1,2,4^, Nathália Costa Almeida Pinho^1,3^, Lucas Vieira Chagas^1,3^, Renan Detoffol Bragança^1,2,4^, Vandack Nobre^1,2,3,4^

#### ^1^NIIMI (Núcleo Interdisciplinar de Investigação em Medicina Intensiva), Belo Horizonte, Brazil; ^2^Hospital das Clinicas da UFMG, Belo Horizonte, Brazil; ^3^Depto Clínica Médica da Faculdade de Medicina da Universidade Federal de Minas Gerais, Belo Horizonte, Brazil; ^4^Programa de Pós-graduação em Infectologia e Medicina Tropical, Universidade Federal de Minas Gerais, Belo Horizonte, Brazil

*Critical Ultrasound Journal 2017,* **9(Supp 1):**A15

**Objective:** Determine in onco-hematology patients with respiratory insufficiency if the utilization of LUS predict the requirement of invasive mechanical ventilation (IMV).

**Methods:** Observational, prospective study. LUS assessment in patients with more than 17 years old admitted to the intensive care unit (ICU) of an University hospital. Four windows were evaluated in each hemithorax, quantifying the aeration loss from 0 to 3 points, namely: 0: A lines; 1: well-defined B lines; 2: coalescent B lines; 3: pulmonary consolidation. The score ranged 0–22.

**Results:** Nine patients were included and 162 videos were performed. The median age was 47 (36–61) years and 55.5% were male. The mortality in ICU was 44.4% and at 28 days was 55.6%. The mean score of LUS at inclusion in patients who required IMV and in those who did not require was 11.4 (± 6) and 2 (± 2.8), respectively (p = 0.07). ROC curve for LUS to predict require IMV at inclusion was 0.96 (p = 0.05; 95% CI 0.83–1.0). Six patients had LUS score at inclusion ≥ 7 and all of them required IMV (p = 0.08). C reactive protein levels, measured at days 1 and 2 after inclusion were significantly higher among patients requiring IMV (p = 0.04).

**Conclusion:** LUS seems to be an useful tool to predict IMV requirement among oncohematological patients. These findings must be confirmed in a larger number of patients.

## A16 Point of care ultrasound in medical graduation

### Maria Thereza Meira Araujo^1^, Luiz Ernani Meira Junior^2^, Luciana Mendes^3^, Jackson Andrade^1^, Nayara Nobre Basso^1^, Anna Cecília Castro e Abreu^1^

#### ^1^Undergraduate Medical Student at Faculdades Integradas Pitágoras, Montes Claros, Brazil; ^2^Department of Emergency Medicine at Faculdades Integradas Pitágoras, Montes Claros, Brazil; ^3^Department of Radiology at Faculdades Integradas Pitágoras, Montes Claros, Brazil

*Critical Ultrasound Journal 2017,* **9(Supp 1):**A16

**Introduction:** Point of care ultrasonography represents a major advance in medical practice as it extends the physical examination of the patient and contributes to a better medical management. However, its use in Brazil is still restricted and can be improved if its teaching is implemented in the basic curriculum of the medical course.

**Objective:** This study aimed to describe the implementation of ultrasound teaching program in medical school in the Faculdades Integradas Pitágoras of Montes Claros.

**Methods:** The sample consisted of 34 medical students from the 11th period who were attending the discipline Urgency and Emergency. Theoretical classes were given in 5 lectures and 5 practical classes about the e-FAST procedure, including the following windows: pericardial window; hepatorenal and hepatodiaphragmatic interfaces; splenorenal and splenodiaphragmatic interfaces; suprapubic; pulmonary and vena cava evaluation. A evaluation form with 64 basic skills for the use of point of care ultrasound was than, applied.

**Results:** The students’ average performance was (96.8 ± 3.6)%. 88.2% of the students had a achieved a result above 95% (> 61 skills correctly identified), noting more difficulty in describing the changes in cardiac tamponade and inferior vena cava.

**Conclusion:** Beyond the need of a future evaluation about the content fixation by the students, we conclude that the teaching of “point of care” ultrasound in the undergraduate course is promising and the students have been able to develop all the basic skills to perform the procedures.

## A17 Ultrasonographic evaluation of the main central venous access points, by medical students

### José Muniz Pazeli Junior, Ana Luisa Silveira Vieira, Bernardo Costa Lemos, Marinna Marques Rodrigues Saliba, Maurício Dutra Costa, Pedro Andrade Mello, Rosimary Souza Vicentino

#### Barbacena’s School of Medicine, Minas Gerais, Brazil

*Critical Ultrasound Journal 2017,* **9(Supp 1):**A17

**Objective:** To demonstrate the simplicity of ultrasound (US) use, the prevalence of anatomical variations of the internal jugular vein (IJV) and evaluate with US visualization the success rate of traditional puncture of IJV, by a simulation.

**Materials and methods:** Five medical students, with no prior US experience, underwent short-term, theoretical-practical, training in US, and then evaluated the IJV and common carotid artery (CCA) of 105 patients. They performed a simulation of the puncture of the IJV, following the anatomical references of the traditional technique (TT), while checking with US if the needle could reach the IJV.

**Results:** The students’s success rate of the US visualization of the IJV and CCA was 95%; the IJV, on the right side, was more commonly found in the anterolateral position in relation to the CCA (38%). On the left side, the most commonly position observed was the anterior (36%). Regarding the IJV caliber in relation to the CCA, a great variability was observed. The success rate in the IJV puncture simulation, observed with US, by the TT was only 55%.

**Conclusion:** There is great variability of the anatomical position and the caliber of the IJV, which reinforces that the ultrasound should be used to guide the puncture of this central vein, reducing the complications.

## A18 Ultrasound guided puncture and central venous access: development of a teaching protocol in animal model

### Anna Cecília Castro e Abreu^1^, Luciana Mendes^2^, Luiz Ernani Meira Junior^3^

#### ^1^Undergraduate Medical Student at Faculdades Integradas Pitágoras, Montes Claros, Brazil; ^2^Department of Radiology at Faculdades Integradas Pitágoras, Montes Claros, Brazil; ^3^Department of Emergency Medicine at Faculdades Integradas Pitágoras, Montes Claros, Brazil

*Critical Ultrasound Journal 2017,* **9(Supp 1):**A18

**Introduction:** The use of ultrasound (US) in clinical practice is an effective method for performing central venous puncture and emergency procedures, being safer than traditional techniques and presenting lower complication rates. In the US, practical US education in medical residency is mandatory in some areas, but in medical graduation there is no curriculum model for its practical and low-cost teaching.

**Objectives:** To develop a low cost didactic protocol for venous access puncture and venous access, using an animal model.

**Methods:** An animal model was developed using thawed raw chicken, olive, cooked quail egg and mushroom (to mimic a nodule to puncture) and a procedure glove with a conducting gel filled phalanx (to mimic a blood vessel) that were inserted in the region between the internal and external animal musculture, through manual dissection. A portable ultrasound device was used and two checklists were developed for the step-by-step direction of the technique.

**Results:** Through the visualization by the US, the animal model was effective in both techniques presenting an ultrasound image similar to the human body.

**Conclusion:** The proposed animal model was effective to reproduce ultrasound images similar to the human image, being possible to train puncture and venous access, showing to be a good model to be reproduced by academic uses.

**Keywords:** Ultrasound, Animal model, Protocol, Medical education

## A19 Applied ultrasonography during the initial assessment of trauma patients

### Fernandez, Juan Pablo; Ahualli, Nicolas; Insfran, Humberto; Fatica, Ivana; Bornia, Jonatan; Denardi, Paula; Algieri, Ruben Daniel

#### Hospital Aeronáutico Central-Fuerza Aérea Argentina, Buenos Aires, Argentina

*Critical Ultrasound Journal 2017,* **9(Supp 1):**A19

**Introduction:** Ultrasonography is regarded as an indispensable element for physicians who work in trauma care and emergency. The training of professionals working in this field is considered as a determinant for the correct decision making in trauma and emergency.

**Objective:** Demonstrate the importance of the use of ultrasonography for the evaluation of the polytraumatized patient

**Materials and methods:** 824 ultrasound exams were performed in a period between June and November of 2016 in which it was evaluated its use in the care of the polytraumatized patient

**Results:** Airway: 135 US. 89 intubation guides (65%). 14 (10.37%) guides for surgical access. Ventilation: closed thoracic trauma. 183 US. Pleural fluid 117 (63.9%) pneumothorax. 53 (28.9%). Trauma open. 97 US. Penetrating wound without pleural lesion: 25 (25.7%)—confirmed by surgery: 11. Pneumothorax: 67 (69.07%). Pneumothorax and pleural fluid: 5 (5.15%). Abdomen: trauma closed: 239 US FAST−: 127 (53.13%) FAST+: 95 (39.7%). FAST− and Vena CAVA Rating: 17 (7.11%). Penetrating trauma: 67 US. FAST− and peritoneal indemnity: 47. Laparoscopic confirmation: 39 (82.9%). FAST+ and peritoneal indemnity: 20 (29.8%) neurological deficit: 83 US. Altered pupil reflex 13 (15.66%) optic nerve altered: 7 (8.4%) deviation cerebral mediated line: 3 (3.6%). Assessment of vascular axes, compartmental syndrome: 145 US. Decreased pulses: 53 (36.5%). Vascular commitment: 17 (11.7%)

**Conclusion:** The incorporation of anatomical and ultrasonographic knowledge favors the quality of care of physicians who perform in extreme situations. The correct use of ultrasonography as a diagnostic tool allows to improve the response times and decision making of surgical procedures.

## A20 Utilization of ultrasonography as a tool to guide for the improvement in the implementation of central venous accesses

### Cristian Flores, Maria Soledad Ferrante, Gustavo Vassia, Carolina Brofman; Victor Ortiz, Rubén Daniel Algieri

#### Hospital Aeronáutico Central-Fuerza Aérea Argentina, Buenos Aires, Argentina

*Critical Ultrasound Journal 2017,* **9(Supp 1):**A20

**Introduction:** The use of ultrasound to perform central venous access allows the recognition of anatomical structures and the constant guide to the success, generating a benefit for the patient and the surgeon.

**Objective:** To determine the usefulness of ultrasonography as a guiding tool in performing surgical procedures.

**Materials and methods:** A prospective descriptive and observational study was carried out. Training through workshops to surgeons in the use of ultrasonography for the identification of anatomical structures and the performance of central venous accesses. The strokes performed in the period from January to June of 2017 were analyzed, using evaluation charts to contemplate the mentioned skills.

**Results:** There were 95 central venous access, all internal jugular venous access; 80 (84.21%) were performed under ultrasound guidance while the remaining 15 (15.78%) were not. Of those made with US, the anatomical structures of the neck were identified in 100% (80). Under ultrasound guidance, 50 (62.5%) required 1 single puncture, 25 (31.5%) 2 punctures and the remaining 5 (6.25%) 3 trials. As for those performed without US 13 (86.66%) were performed with 3 punctures and the remaining 2 (13.33%) with more than 3. It was evident in those performed with US a considerable reduction of surgical times.

**Conclusion:** The use of ultrasonography as a guiding tool for central venous access allows improving the efficiency of procedures, improving the quality of care.

## A21 Emergency bedside ultrasound in first trimester pregnancy; knowledge, attitudes and practices survey of documentation and reimbursement

### Elizabeth Krebs, Frances Shofer, Cameron Baston, Christy Moore, Wilma Chan, Anthony J. Dean, Nova Panebianco

#### Division of Ultrasound, Department of Emergency Medicine, University of Pennsylvania, Philadelphia, PA, USA

*Critical Ultrasound Journal 2017,* **9(Supp 1):**A21

**Study objectives:** Emergency ultrasound (EMBU) improves clinical decision-making, patient satisfaction, and time to disposition. This study investigates physicians’ knowledge, attitudes and practices about performance and documentation of EMBU to confirm intrauterine pregnancy (IUP) in Emergency Department (ED) patients at risk for ectopic pregnancy.

**Methods:** An anonymous, web-based survey was sent to attending and resident physicians in an academic, urban ED with an annual census of 65,000. Twenty EMBU questions evaluated respondents: experience (years), comfort level, knowledge about reimbursement, documentation requirements and preferred compliance prompting.

**Results:** Fifty-nine of 98 physicians (60%, 26/52 attending and 33/46 residents) reported 0–20 years’ EMBU experience (median 4). Mean scores (0–100, uncomfortable to completely comfortable) for EMBU performance and interpretation were 81 (SD 23) and 80 (SD 18) respectively. Ninety percent agree EMBU enhances patient care; 92% that it accelerates disposition and diagnoses and 88% that patients appreciate it. Twenty-three percent are unaware that EMBU is a billable procedure that can generate ED revenue. Inadequate documentation methods were reported by 54% but 49% assert they use a template designed for billing compliance. Thirty-seven percent report that Electronic Medical Record (EMR) prompts may improve documentation compliance while 64% appreciate sonographer-educator presence.

**Conclusion:** Most physicians are comfortable with EMBU for IUP and recognize the clinical benefits. Reimbursement and documentation knowledge is less prevalent. Education, promoting documentation templates and sonographer-educator support may improve documentation and reimbursement in the future.

## A22 Middle cerebral and common carotid arteries color-Doppler evaluation during cardiopulmonary resuscitation

### Stefano Geniere Nigra^1^, Carmela Graci^2^, Vito Sgromo^3^, Alberto Casazza^4^, Giacomo Veronese^5^, Miguel Montorfano^6^, Giovanni Ricevuti^1^

#### ^1^University of Pavia, Pavia, Italy; ^2^AREU-Azienda Regionale Emergenza Urgenza, Niguarda Hospital, Milan, Italy; ^3^AREU-Azienda Regionale Emergenza Urgenza, Policlinico San Matteo Hospital, Pavia, Italy; ^4^ASST-Azienda Socio-Sanitaria Terrritoriale Pavia-Ospedale Civile, Vigevano, Italy; ^5^Emergency Medicine Resident, University of Bicocca, Milan, Italy; ^6^Department of Ultrasound, Hospital de Emergencias “Dr. Clemente Álvarez” (HECA), Rosario, Argentina

*Critical Ultrasound Journal 2017,* **9(Supp 1):**A22

**Background:** Cardiac arrest (CA) is one of the major causes of death worldwide. Mortality is improving but neurological prognosis remains bad. Cerebral hypoperfusion and hypoxia are the main factors determining neurological outcome. Adequate cerebral perfusion is one of the main therapeutic goals and it is mainly based on high quality CPR. Unfortunately, there are no validated methods to monitor chest compression performance and effective cerebral blood flow during CPR.

**Study design:** Prospective cohort study.

**Objective:** MCA and CCA evaluation (peak systolic [Vs] & end-diastolic [Vd] velocity, pulsatility index [PI]) during CPR (manual vs mechanical); relationship between MCA and CCA patterns and neurological outcome using Cerebral Performance Categories scale.

**Methods:** Inclusion criteria: all patients with non-traumatic CA undergoing CPR. After clinical diagnosis of CA, beginning of CPR and airways management, we perform MCA and CCA color Doppler evaluation. Examination is repeated, whenever possible, during all CPR steps and if any clinical condition variations (i.e. ROSC, death) occur.

**Results:** we describe a case of patient underwent sudden CA. MCA and CCA evaluation showed forward-flow during compression and back-flow pattern during chest release; during CPR, MCA profile was brain death-like pattern; during clinical pulse check evaluation (PEA, no pulse), anterograde flow pattern was registered with very poor velocities. When ROSC occurred, hyperemic MCA pattern was obtained. After 3 days the patient was awake with no brain damage.

**Conclusion:** Color Doppler monitoring may provide information on CPR quality and it might be useful for early detection of ROSC and prognostic stratification of patients with CA.

## A23 Priapism: point of care Doppler ultrasound aspects

### Marina Marazzi, María Fernanda Barbui, Gabriela Da Campo, Miguel Montorfano

#### Department of Ultrasound and Doppler, Hospital de Emergencias “Dr. Clemente Álvarez” (HECA), Avenida Pellegrini 3205, Rosario, Argentina

*Critical Ultrasound Journal 2017,* **9(Supp 1):**A23

**Background:** Priapism is a prolonged, pathologic erection, lasting more than 4 h. There are 2 main types: ischemic or low-flow priapism and non-ischemic or high-flow priapism. Ischemic type is an emergency. If untreated can lead to permanent damage, corporal fibrosis and potential erectile disfunction. Color and pulse Doppler can help in the rapid differentiation of these two types of priapism.

**Objective:** To present 3 cases and review the utility of Doppler ultrasound in the triage of patients with priapism.

**Cases series:** 3 patients with priapism were evaluated in August 2017: 2 were caused by the use of non legal drugs and one occurred after an instrumentation of the urethra. All the 3 patients present low flow ischemic priapism and immediate treatment was required.

**Discussion:** Doppler ultrasound can be useful in the differentiation of high vs low flow priapism. Patients with low-flow priapism can have thrombosis of the corpora cavernosa or corpus spongiosum and decreased or absent of color flow or spectral Doppler in the cavernosal arteries with increased resistance Index if flow is present. Flow in the superficial dorsal vein and increase resistance index in the dorsal artery may be present. Low flow priapism represents 95% of the cases. Patients with high flow priapism can have normal or elevated arterial Doppler velocities. Flow suggesting arteriovenous flow fistula may be present. Usually occurs after a local trauma.

**Conclusion:** Doppler ultrasound is a rapid method to distinguish between low flow and high flow priapism and helps in the acute decision making process.

## A24 Point of care ultrasound in the initial evaluation and triage of scrotal trauma

### Maria Fernanda Barbui, Marina Marazzi, Gabriela Da Campo, Cecilia Ciarlo, Leonardo Vera, Miguel Montorfano

#### Department of Ultrasound and Doppler, Hospital de Emergencias “Dr. Clemente Álvarez” (HECA), Avenida Pellegrini 3205, Rosario, Argentina

*Critical Ultrasound Journal 2017,* **9(Supp 1):**A24

**Background:** Scrotal trauma represents less than 1% of all trauma injuries. Ultrasound is an essential tool for the initial evaluation of these patients. It permits to rapidly diagnose the presence of hydrocele, haematocele, intra or extra testicular hematoma, contusion and disruption of the tunica albuginea.

**Objective:** Evaluate the utility of ultrasound in the triage of patients for medical or surgical management.

**Methods:** A prospective observational study between January 2015 and February 2017 was performed. 13 patients were evaluated: 9 with penetrating trauma (gunshot wound) and 4 with blunt trauma (1 caused by fall of height and 3 caused by a road traffic accident)

**Results:** In 8 patients (7 with penetrating wounds and 1 with blunt trauma) US showed diffuse heterogeneous texture, contour irregularity and disruption of the tunica albuginea. 5 patients (3 with penetrating wounds and 2 with blunt trauma) had normal US aspect (2), small amount of peritesticular fluid (1), and small intratesticular hematoma (2)

All the 8 patients with diffuse heterogeneous testicular parenchyma, contour irregularity and disruption of the tunica albugínea were sent to the OR: testicular rupture was confirmed in all cases. The other 5 patients underwent medical observation and ultrasound follow up with ultrasound until the complete resolution (sensitivity and specificity of 100% for the diagnosis of testicular rupture).

**Conclusions:** US is a very sensitive and specific tool for the selection of patients with testicular rupture that require immediate surgical exploration.

## A25 Point of care ultrasound (POCUS) evaluating an unusual case of edematous-ascitic syndrome (EAS)

### Matías Brizuela, Mariana Lía Brizuela, Marcos Aqcuavita, Javier Buchanan, José Alejandro Bujedo

#### Unidad de Terapia Intensiva, Sanatorio Privado del Interior S.R.L., Rio Ceballos, Córdoba, Argentina

*Critical Ultrasound Journal 2017,* **9(Supp 1):**A25

**Objective:** To describe the multifaceted use of POCUS in the evaluation of a patient with EAS.

**Case report:** A 70-year-old man was transferred to critical care unit suspected of having decompensated heart failure. History of arterial hypertension, ischemic heart disease, atrial fibrillation (AF) and chronic renal failure. Ex-alcoholic drinker and severe smoker. Stable hemodynamically, tricuspid focus regurgitation murmur, lower limb edema, tension ascites and bilateral pleural effusion. Bedside ultrasonography evidenced dilatation of right cavities, 2 echogenic masses occupying part of the right atrium and ventricle, non-mobile, rounded, regular suggestive of thrombus vs. tumors. Moderate pulmonary hypertension. Laminar pericardial effusion. Absence of deep venous thrombosis. Bilateral pleural effusion, bilateral B lines. Liver with 2 irregular and vascularized masses occupying the right lobe, right adrenal gland nodule, splenomegaly, small kidneys, stones in the gallbladder and important ascites. 4000 ml of ascitic fluid was evacuated under ultrasound guidance, Albumin Gradient Suero-Ascites = 1.8. Anticoagulation was started with intravenous unfractionated heparin for 5 days and then continued with enoxaparin. Tomography scan also showed mediastinal and retroperitoneal adenomegalies, and presence of nodular image in the lower left lobe of the lung. Alpha-fetoprotein 19.9 UI/ml, serology virus human immunodeficiency, hepatitis B and C negative. Ascitic fluid was negative for malignancy. Due to associated comorbidities surgical management was ruled out and hepatic biopsy was planned. During hospitalization he presented sudden death.

**Conclusion:** POCUS provided diagnosis of possible etiologies and complications and served as guided of invasive procedures.

**Consent for publication:** The authors confirm that written informed consent was obtained for publication.

## A26 Supraclavicular (SC) ultrasound-guided (USG) catheterization of the brachiocephalic vein in infants and children

### Pablo Bravo Figueroa^1^, V. Ricardo Carvajal^1^, P. Oscar Bravo^1^, N. Monserrat Navarro^1^, J. Rodrigo Adasme^2^

#### ^1^Unidad Paciente Crítico Pediátrico, Hospital San Juan de Dios, Santiago, Chile; ^2^Respiratory Therapy Unit UC, Magister in Epidemiology, University of Los Andes, Las Condes, Chile

*Critical Ultrasound Journal 2017,* **9(Supp 1):**A26

**Introduction:** Critical patients often require a central venous catheter (CVC), there is experience in USG cannulation of the jugular and femoral vein. But there is a group of patients who, due to their previous condition, multiple punctures, hypovolemic status, or presence of tracheotomy, the cannulation of these EG accesses is difficult.

**Objectives:** Retrospective SC USG catheterization of brachicephalic vein between 2013 and 2017.

**Materials:** Patients with SC USG catheterization of the brachiocephalic vein from March 2013 to July 2017 installed in Pediatric Critical Care Unit.

**Results:** A total of 94 procedures were analyzed: 47% were younger than 1 year, female gender predominated (54%), 30% were under 5 kilos; Main diagnoses: Low Respiratory Infection and Nephro-Urological Pathologies (34 and 16%); 25% of the patients had tracheotomy. Thirty-seven percent of the patients did not require invasive airway intervention. The most frequently chosen side was the left side (57%), the vast majority was achieved at the first attempt by 77%, with a total success rate of 99%. There was an arterial puncture.

**Comments:** SC USG catheterization of the brachiocephalic vein in critical pediatric patients is a safe, rapid technique with few complications. Very useful in small patients, in spontaneous breathing, with tracheotomy and in non-invasive mechanical ventilation.

## A27 Ultrasound-guided central venous cannulation in Preterm of very low weight (< 1500 g)

### Pablo Bravo Figueroa^1^, Carolina Méndez^2^, Rodrigo Adasme J.^3^

#### ^1^Pediatric Intensivist, Pediatric Critical Patient Unit, San Juan de Dios Hospital, Santiago, Chile; ^2^Neonatology Service, San Juan de Dios Hospital, Santiago, Chile; ^3^Respiratory Therapy Unit UC, Magister in Epidemiology, University of Los Andes, Las Condes, Chile

*Critical Ultrasound Journal 2017,* **9(Supp 1):**A27

**Introduction:** CVC echo-guided puncture has improved the success rate and decreased mechanical complications. The central accesses in vessels of the neck without ultrasound in this group is not an alternative due to the complications given the size of structures and the difficulty of classic anatomical repairs.

**Objectives:** Demonstrate Efficacy and Safety in transient echo-guided CVC installation in premature infants less than 1500 g hospitalized in the Neonatology Unit of our Hospital (NICU).

**Materials:** Retrospective review of 2 cohorts (< 1500 and > 1500 g). Transient central venous catheters installed in the NICU between March 2015 and July 2017 guided echoes. Statistical analysis of both cohorts (NBFR < 1500 and NBST > 1500) was performed: the eleventh hypothesis was performed with Fisher’s exact test, Wilcoxon Mann–Whitney test or Student’s T according to variable distribution comparing less than 1500 g and 1500 or more grams, with a significance level of p < 0.05; to assess risk of increased punctures, they were moderated under Poisson regression with incidence risk ratio (IRR), 95% confidence interval and p value, performing univariate for each independent variable recorded.

**Results:** There were 42 procedures with a median age of 25 days, with a median weight of 1962 g: range of 677–4500 g. There were 2 frustoced procedures (1 in each group) Overall success rate 61.9, 31 and 2.4% at the first, second and third attempt. There was an arterial puncture as a complication. When comparing both groups there was no statistical difference there was no difference in numbers of punctures in achieving access nor in complications.

**Comments:** The use of ultrasound allows channeling in PTNB < 1500 g with a high success rate (95.3%). The results are similar to those described in the literature. This technique is safe and effective for professionals with CVC eco-guided experience

## A28 Poor correlation between IVC variability and haematocrit level to determine intravascular volume status in spontaneous breathing adult with dengue fever

### Adi Osman^1^, Azma Haryaty Ahmad^1^, Seri Rohayu Neow Hanzah^1^, Emilia Mohtar Razali^2^

#### ^1^Department of Trauma and Emergency Medicine, Raja Permaisuri Bainun Hospital, 30450, Ipoh, Perak, Malaysia; ^2^Department of Trauma and Emergency Medicine, National University Hospital, Cheras, 56000, Kuala Lumpur, Malaysia

*Critical Ultrasound Journal 2017,* **9(Supp 1):**A28

**Objectives:** Detection of intravascular depletion is paramount in early assessment of DF. We aimed to focus on the value of Inferior Vena Cava (IVC) variability as an intravascular volume assessment in spontaneously breathing adult dengue patient with or without warning signs in correlation with haematocrit (HCT) level.

**Methods:** This was a single centre prospective cross-sectional study. The primary outcome was to measure the inferior vena cava variability in correlation with haematocrit level in predicting the intravascular volume status in DF with or without warning signs. The secondary outcome was the survival and complications after 24 h.

**Results:** Two hundred and two dengue patients were analyzed. There was a poor correlation between diameter of IVC during expiration (IVCe), IVC during inspiration (IVCi) and IVC collapsibility index (IVCci) with HCT level in patients with and without warning signs (r = − 0.251, − 0.268 and 0.209 respectively) with p < 0.01. IVCe and IVCi had a significant negative correlation in DF with warning signs (r = − 0.369 and − 0.415 respectively) with p < 0.01. And zero correlation between IVCe and IVCi with HCT level in DF without warning signs (p > 0.5). No adverse effects were recorded within the extrapolated time.

**Conclusion:** Our study demonstrates poor relationship between IVC diameter and HCT level in DF. The usage of IVC variability in assessing intravascular volume status in haemodynamically stable DF patients with or without warning sign can be misleading.

